# Supplementary material for: Spin–orbit coupled molecular quantum magnetism realized in inorganic solid
Source: Nat Commun. 2016 Sep 21;7:12912. doi: 10.1038/ncomms12912 (PMC5035996; doi:10.1038/ncomms12912)
Supplement: Supplementary Information — Supplementary Figures 1-6, Supplementary Table 1, Supplementary Notes 1-4 and Supplementary References. [file ncomms12912-s1.pdf]

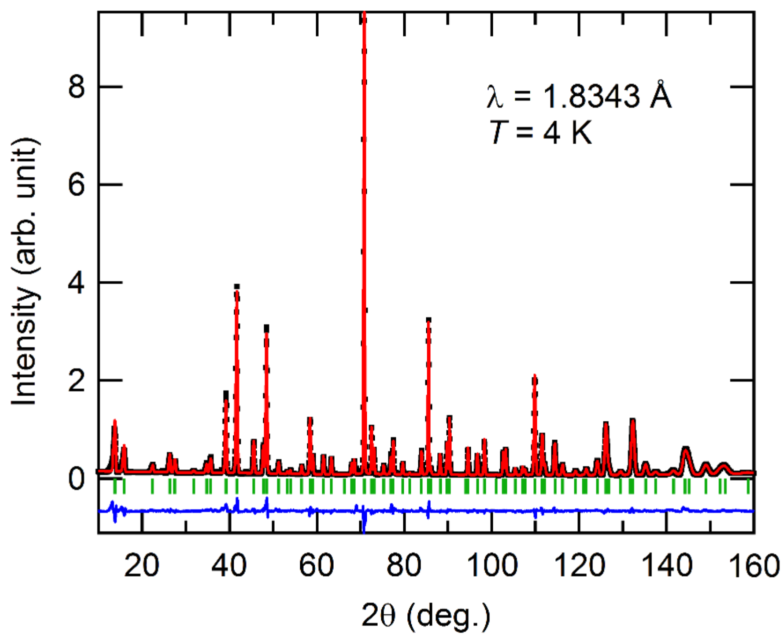


Supplementary Figure 1. Neutron powder diffraction pattern at 4 K. Circles are the data and green bars denote nuclear Bragg peak positions. The black line represents the calculated intensity and difference between data and calculation, respectively.

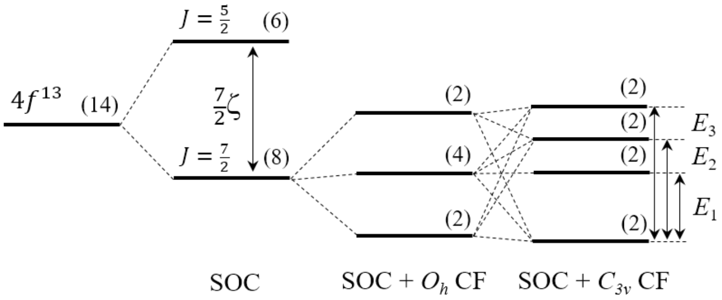


Supplementary Figure 2. Schematic crystal field (CF) energy splitting diagram of one hole ($4f^{13}$) states with spin orbit coupling (SOC), SOC + cubic $O_h$ symmetry CF, and SOC + trigonal $C_{3v}$ symmetry CF.

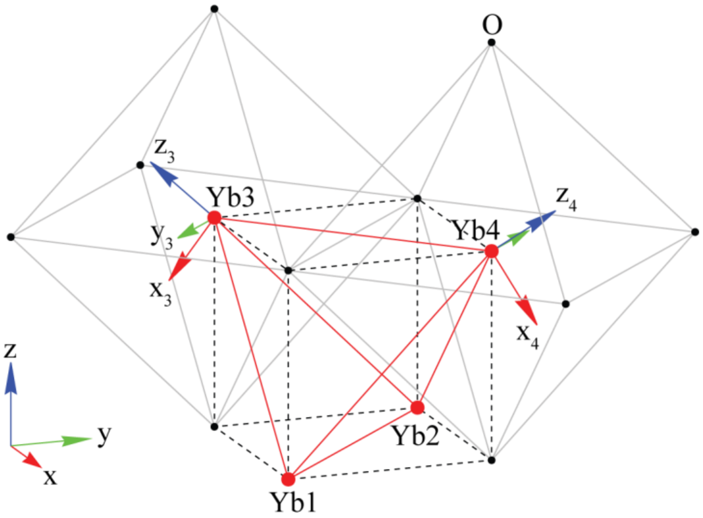


Supplementary Figure 3. Schematic diagram of edge-shared $Yb^{3+}$ ions in a $Yb_4$-tetrahedron. Yb and O ions are presented by red and black spheres, respectively. The Yb ions locate at (1 0 0), (0 1 0), (0 0 1), and (1 1 1) in the (**x**, **y**, **z**)-global coordinates. The local coordinates centered at the *i*-th Yb site is presented with ($\mathbf{x}_i$, $\mathbf{y}_i$, $\mathbf{z}_i$).

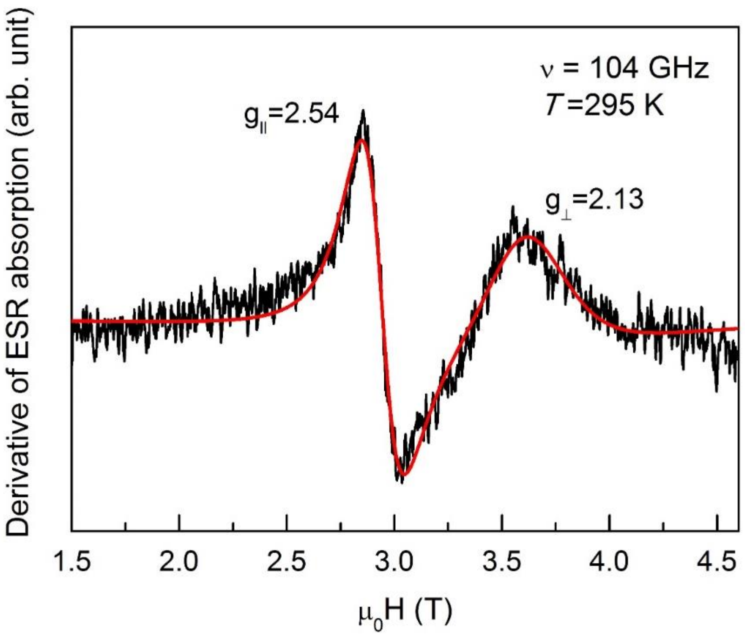


Supplementary Figure 4. Derivative of the EPR absorption of polycrystalline $Ba_3Yb_2Zn_5O_{11}$ sample measured at *v* = 104 GHz and *T* = 295 K. The solid red line is a fit to a sum of two Lorentzian profiles.

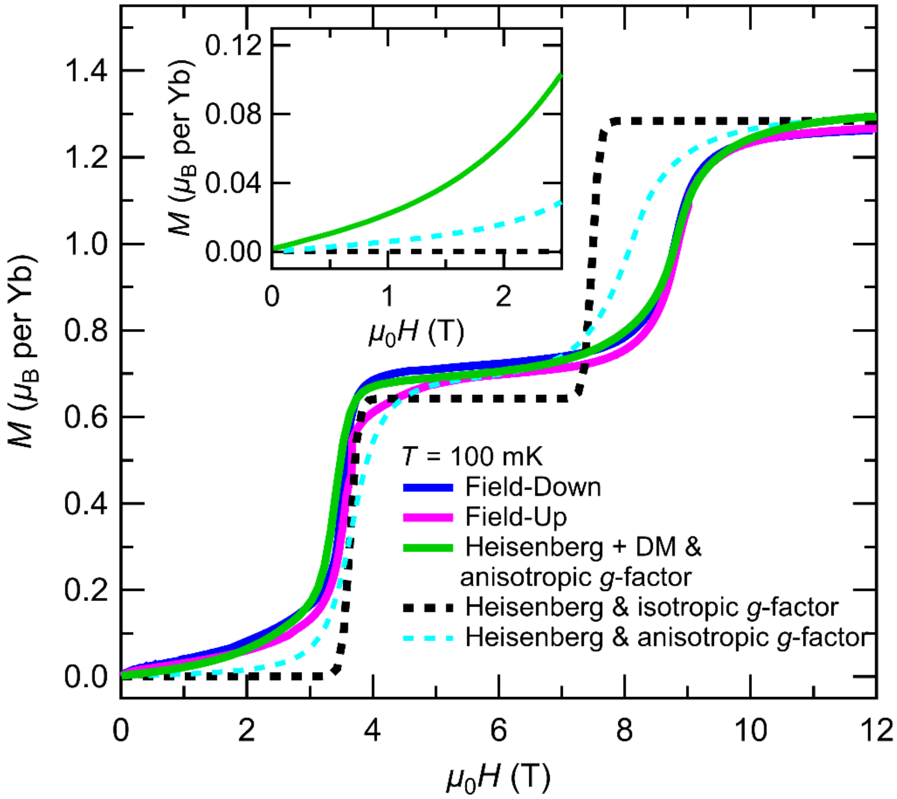


Supplementary Figure 5. The measured and simulated magnetization $M(H)$. The magenta and blue solid lines are the measured $M(H)$ with 7.5 mT·min$^{-1}$ sweep-rate for up- and down-field, respectively. The green solid line is the simulated magnetization $M(H)$ from $\mathcal{H}_{\mathrm{eff}}$ with $J$ = 0.589 meV, $d/J$ = 0.27, $g_{\parallel} = 3.0$ and $g_{\perp} = 2.4$. The black and cyan dashed lines are simulated from the conventional Heisenberg Hamiltonian (no DM term) with an isotropic $g$-factor ($g$ = 2.569) and anisotropic $g$-factors ( $g_{\parallel} = 3.0$ and $g_{\perp} = 2.4$ ), respectively. The inset shows magnified low field region of the simulated $M(H)$, evincing the paramagnetic response of three models near the zero field.

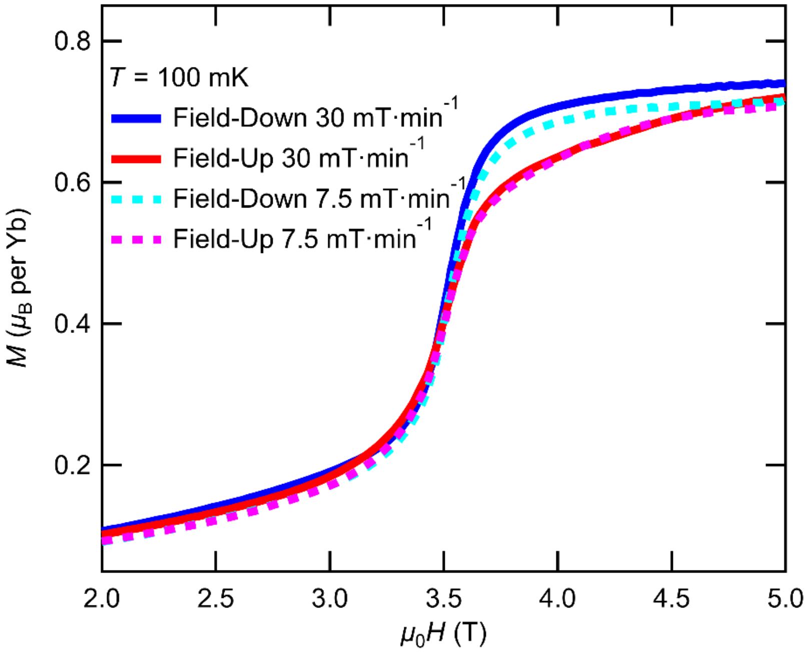


Supplementary Figure 6. Observed $M(H)$ around $H_{C1}$ with different field sweep-rate at $T$ = 100 mK. Solid and dashed lines denote $M(H)$'s at the field sweep rate of 30 mT·min$^{-1}$ and 7.5 mT·min$^{-1}$, respectively. Hysteretic behavior is enhanced at the faster field sweep rate.

Supplementary Table 1. Crystallographic data and details of data refinement

| | |
|---|---|
| Formula | $Ba_3Yb_2Zn_5O_{11}$ |
| Space Group | $F\bar{4}3m$ |
| Formula weight (g/mol) | 1260.978 |
| Density (g/cm$^3$) | 6.861 |
| a, b, c (Å) | 13.46594(7), 13.46594(7), 13.46594(7) |
| α, β, γ (°) | 90, 90, 90 |
| *V* (Å$^3$) | 2441.80(4) |
| Temperature (K) | 4 |
| Neutron Wavelength (Å) | 1.8343 |
| 2θ range | 7.5 – 159.0 |
| No. Reflections | 111 |
| $\chi^2$ | 14.1 |
| $R_p$ | 0.097 |
| $R_{wp}$ | 0.114 |
| $R_B$ | 0.042 |
| $R_F$ | 0.025 |
| Atoms | |
| Ba | |
| Site, x, $B_{iso}$ | 24*f*, 0.7059(3), 0.79(6) |
| Yb | |
| Site, x, $B_{iso}$ | 16*e*, 0.8365(1), 0.78(3) |
| Zn1 | |
| Site, x, $B_{iso}$ | 16*e*, 0.0832(2), 1.23(7) |
| Zn2 | |
| Site, z, $B_{iso}$ | 24*g*, 0.0832(3), 1.15(5) |
| O1 | |
| Site, $B_{iso}$ | 4*b*, 1.2(1) |
| O2 | |
| Site, x, $B_{iso}$ | 16*e*, 0.3422(2), 1.07(7) |
| O3 | |
| Site, $B_{iso}$ | 4*a*, 1.0(1) |
| O4 | |
| Site, x, $B_{iso}$ | 16*e*, 0.6673(3), 1.29(6) |
| O5 | |
| Site, x, z, $B_{iso}$ | 48*h*, 0.1661(1), 0.9999(2), 1.13(4) |

## Supplementary Note 1. Effective Hamiltonian and inelastic neutron scattering

The Yb ion in $Ba_3Yb_2Zn_5O_{11}$ is surrounded by six oxygens, i.e. $YbO_6$ octahedron, and four neighboring Yb ions form an isolated tetrahedron. The octahedron is slightly distorted in a space group $F\bar{4}3m$ . Each Yb site in the isolated tetrahedron is under a $C_{3\mathrm{v}}$ local symmetry and the threefold axes are located along the [1 -1 -1], [-1 1 -1], [-1 -1 1], and [1 1 1] directions as can be seen in Fig. 1c. Due to the strong spin-orbit coupling, the ground state of the $Yb^{3+}$ ($4f^{13}$) ion is the total angular momentum $j$ = 7/2 state under a spherical symmetry with a local quantization axis corresponding to one of threefold axes. The eight-fold $j$ = 7/2 state is split into four Kramers doublets (see Supplementary Fig. 2) under the $C_{3\mathrm{v}}$ symmetry, and the highest energy Kramers doublet states can be expressed by,

$$|\pm\rangle_{z_i} = \mp C_1\left|\tfrac{7}{2},\mp\tfrac{7}{2}\right\rangle_{z_i} - C_2\left|\tfrac{7}{2},\mp\tfrac{1}{2}\right\rangle_{z_i} \pm C_3\left|\tfrac{7}{2},\pm\tfrac{5}{2}\right\rangle_{z_i}. \tag{1}$$

Here, $\left|\tfrac{7}{2},m\right\rangle_{z_i}$ is a state with $j$ = 7/2 and $j_{z_i}$ = m, and $z_i$ is the local quantization axis at the $i$-th site in a tetrahedron (see Supplementary Fig. 3).

The $Yb^{3+}$ ($4f^{13}$) ground state corresponds to one electron removal state at this Kramers doublet from the fully occupied $4f^{14}$ state [1]. The doublet ground state is energetically separated from the first excited state by 38.2 meV (= 443 K·$k_B$) [2]. As the results, the $Yb^{3+}$ magnetic spin effectively has a pseudospin-1/2 with a mixture of spin 1/2 and -1/2 states. As the results, the magnetic interaction should be described by a generalized magnetic exchange Hamiltonian with tensor forms of the exchange coupling tensor $\mathbf{J}'$and $g$-tensor $\mathbf{g}'$ in the local coordinates,

$$\mathcal{H}_{\mathrm{gen}} = \sum_{i<j} \mathbf{S}_i \cdot \mathbf{J}'_{\boldsymbol{ij}} \cdot \mathbf{S}_j - \mu_B \mathbf{H} \cdot \sum_i \mathbf{g}'_i \cdot \mathbf{S}_i\,. \tag{2}$$

The doublet ground state can be rewritten as $|D_\sigma\rangle_{z_i} = -\sigma c_{i,-\sigma}|FO\rangle$ with the pseudospin 1/2 states denoted by $\sigma(=\pm)$. Here $c_{i,-\sigma}$ is the one electron annihilation operator to reduce to the $|-\sigma\rangle_{z_i}$ state. In this notation, the pseudospin 1/2 operators $\mathbf{S}_i$, which correspond to the Pauli spin operators on the subspace of the Kramers doublet at the $i$-th site, are given by

$$S_{i,x_i} = \frac{1}{2}\left(|D_+\rangle_{z_i\,z_i}\langle D_-| + |D_-\rangle_{z_i\,z_i}\langle D_+|\right),$$

$$S_{i,y_i} = \frac{1}{2i}\left(|D_+\rangle_{z_i\,z_i}\langle D_-| - |D_-\rangle_{z_i\,z_i}\langle D_+|\right), \text{ and}$$

$$S_{i,z_i} = \frac{1}{2}\left(|D_+\rangle_{z_i\,z_i}\langle D_+| - |D_-\rangle_{z_i\,z_i}\langle D_-|\right). \tag{3}$$

Now the generalized magnetic Hamiltonian has a matrix form of

$$\mathcal{H}_{\mathrm{gen}} = \sum_{i<j} J'^{\mu\nu}_{ij} S^\mu_i S^\nu_j - \mu_B H^\mu \sum_i g'^{\mu\nu}_i S^\nu_i\,. \tag{4}$$

$J'^{\mu\nu}_{ij}$ and $g'^{\mu\nu}_i$ are site independent and respectively expressed as

$$J'^{\mu\nu} = \begin{pmatrix} \mathcal{J}'_{xx} & \mathcal{J}'_{xy} & \mathcal{J}'_{xz} \\ \mathcal{J}'_{yx} & \mathcal{J}'_{yy} & \mathcal{J}'_{yz} \\ \mathcal{J}'_{zx} & \mathcal{J}'_{zy} & \mathcal{J}'_{zz} \end{pmatrix} \text{ and } g'^{\mu\nu} = \begin{pmatrix} g_\perp & 0 & 0 \\ 0 & g_\perp & 0 \\ 0 & 0 & g_\parallel \end{pmatrix}. \quad (5)$$

The local magnetic moment operator $\mathbf{M}_i$ is equivalent to $g_\perp\left(S_{i,x_i}\hat{\mathbf{x}}_i - S_{i,y_i}\hat{\mathbf{y}}_i\right) + g_\parallel S_{i,z_i}\hat{\mathbf{z}}_i$ where $g_\perp = 2\,{}_{z_i}\langle D_+|M_{i,x_i}|D_-\rangle_{z_i} = -2i\,{}_{z_i}\langle D_+|M_{i,y_i}|D_-\rangle_{z_i}$ and $g_\parallel = 2\,{}_{z_i}\langle D_+|M_{i,z_i}|D_+\rangle_{z_i}$. It is noticed that the total angular momentum operators can be also expressed with the pseudospin operators like $\mathbf{J}_i = \alpha_\perp\left(S_{i,x_i}\hat{\mathbf{x}}_i - S_{i,y_i}\hat{\mathbf{y}}_i\right) + \alpha_\parallel S_{i,z_i}\hat{\mathbf{z}}_i$ where $\alpha_\perp = 2\,{}_{z_i}\langle D_+|\mathrm{J}_{i,x_i}|D_-\rangle_{z_i} = g_\mathrm{J} g_\perp$, $\alpha_\parallel = 2\,{}_{z_i}\langle D_+|\mathrm{J}_{i,z_i}|D_+\rangle_{z_i} = g_\mathrm{J} g_\parallel$, and the Landé $g$-factor $g_\mathrm{J} = 8/7$.

Now $\mathcal{H}_\mathrm{gen}$ can be expressed in the global coordinates by defining transform matrices $\mathbf{Q}_{z_i}$ under the $T_\mathrm{d}$ symmetry as followings,

$$\mathbf{Q}_{z_1} = \begin{pmatrix} \frac{1}{\sqrt{6}} & -\frac{1}{\sqrt{6}} & \frac{2}{\sqrt{6}} \\ -\frac{1}{\sqrt{2}} & -\frac{1}{\sqrt{2}} & 0 \\ \frac{1}{\sqrt{3}} & -\frac{1}{\sqrt{3}} & -\frac{1}{\sqrt{3}} \end{pmatrix}, \mathbf{Q}_{z_2} = \begin{pmatrix} -\frac{1}{\sqrt{6}} & \frac{1}{\sqrt{6}} & \frac{2}{\sqrt{6}} \\ \frac{1}{\sqrt{2}} & \frac{1}{\sqrt{2}} & 0 \\ -\frac{1}{\sqrt{3}} & \frac{1}{\sqrt{3}} & -\frac{1}{\sqrt{3}} \end{pmatrix},$$

$$\mathbf{Q}_{z_3} = \begin{pmatrix} -\frac{1}{\sqrt{6}} & -\frac{1}{\sqrt{6}} & -\frac{2}{\sqrt{6}} \\ \frac{1}{\sqrt{2}} & -\frac{1}{\sqrt{2}} & 0 \\ -\frac{1}{\sqrt{3}} & -\frac{1}{\sqrt{3}} & \frac{1}{\sqrt{3}} \end{pmatrix}, \mathbf{Q}_{z_4} = \begin{pmatrix} \frac{1}{\sqrt{6}} & \frac{1}{\sqrt{6}} & -\frac{2}{\sqrt{6}} \\ -\frac{1}{\sqrt{2}} & \frac{1}{\sqrt{2}} & 0 \\ \frac{1}{\sqrt{3}} & \frac{1}{\sqrt{3}} & \frac{1}{\sqrt{3}} \end{pmatrix}. \quad (6)$$

The transform matrices $\mathbf{Q}_{z_i}$ transform local Cartesian coordinate $\hat{\mathbf{x}}_\mathrm{i}$, $\mathbf{J}'$, and $\mathbf{g}'$ as

$\hat{\mathbf{x}} = \mathbf{Q}_{z_i}^\mathrm{T}\hat{\mathbf{x}}_i$, $\mathbf{J}_{ij} = \mathbf{Q}_{z_i}^\mathrm{T}\mathbf{J}'\mathbf{Q}_{z_j}$, and $\mathbf{g}_i = \mathbf{Q}_{z_i}^\mathrm{T}\mathbf{g}'\mathbf{Q}_{z_i}$, respectively.

Then the generalized Hamiltonian is represented with the transformed exchange coupling $\mathbf{J}_{ij}$ and $g$-factor $\mathbf{g}_i$ in the global coordinates as

$$\mathcal{H}_\mathrm{gen} = \sum_{i<j}\mathcal{H}_{ij} - \mu_B\mathbf{H}\cdot\sum_i \mathbf{g}_i\cdot\mathbf{S}_i\,, \quad (7)$$

where $\mathcal{H}_{ij} = \mathbf{S}_i\cdot\mathbf{J}_{ij}\cdot\mathbf{S}_j$.

The matrix elements of $\mathbf{J}_{ij}$ and $\mathbf{g}_i$ can respectively be expressed in terms of four $\mathcal{J}_i$ ($i = 1, 2, 3, \text{and } 4$) parameters [3, 4] and two parameters $g_d = (g_\parallel + 2g_\perp)/3$ and $g_o = (g_\parallel - g_\perp)/3$ as followings;

$$\mathbf{J}_{12} = \begin{pmatrix} \mathcal{J}_1 & -\mathcal{J}_3 & -\mathcal{J}_4 \\ -\mathcal{J}_3 & \mathcal{J}_1 & \mathcal{J}_4 \\ \mathcal{J}_4 & -\mathcal{J}_4 & \mathcal{J}_2 \end{pmatrix}, \mathbf{J}_{13} = \begin{pmatrix} \mathcal{J}_1 & -\mathcal{J}_4 & -\mathcal{J}_3 \\ \mathcal{J}_4 & \mathcal{J}_2 & -\mathcal{J}_4 \\ -\mathcal{J}_3 & \mathcal{J}_4 & \mathcal{J}_1 \end{pmatrix}, \mathbf{J}_{14} = \begin{pmatrix} \mathcal{J}_2 & \mathcal{J}_4 & \mathcal{J}_4 \\ -\mathcal{J}_4 & \mathcal{J}_1 & \mathcal{J}_3 \\ -\mathcal{J}_4 & \mathcal{J}_3 & \mathcal{J}_1 \end{pmatrix},$$

$$\mathbf{J}_{23} = \begin{pmatrix} \mathcal{J}_2 & \mathcal{J}_4 & -\mathcal{J}_4 \\ -\mathcal{J}_4 & \mathcal{J}_1 & -\mathcal{J}_3 \\ \mathcal{J}_4 & -\mathcal{J}_3 & \mathcal{J}_1 \end{pmatrix}, \mathbf{J}_{24} = \begin{pmatrix} \mathcal{J}_1 & -\mathcal{J}_4 & \mathcal{J}_3 \\ \mathcal{J}_4 & \mathcal{J}_2 & \mathcal{J}_4 \\ \mathcal{J}_3 & -\mathcal{J}_4 & \mathcal{J}_1 \end{pmatrix}, \mathbf{J}_{34} = \begin{pmatrix} \mathcal{J}_1 & \mathcal{J}_3 & -\mathcal{J}_4 \\ \mathcal{J}_3 & \mathcal{J}_1 & -\mathcal{J}_4 \\ \mathcal{J}_4 & \mathcal{J}_4 & \mathcal{J}_2 \end{pmatrix}, \quad (8)$$

$$\mathbf{g}_1 = \begin{pmatrix} g_d & -g_o & -g_o \\ -g_o & g_d & g_o \\ -g_o & g_o & g_d \end{pmatrix}, \mathbf{g}_2 = \begin{pmatrix} g_d & -g_o & g_o \\ -g_o & g_d & -g_o \\ g_o & -g_o & g_d \end{pmatrix},$$

$$\mathbf{g}_3 = \begin{pmatrix} g_d & g_o & -g_o \\ g_o & g_d & -g_o \\ -g_o & -g_o & g_d \end{pmatrix}, \mathbf{g}_4 = \begin{pmatrix} g_d & g_o & g_o \\ g_o & g_d & g_o \\ g_o & g_o & g_d \end{pmatrix}. \tag{9}$$

It is worth to note that $\mathcal{H}_{ij}$ can always be decomposed into the Heisenberg, Dzyaloshinskii-Moriya (DM), and pseudodipolar interactions, which corresponds to the 0th, 1st, and 2nd rank tensors, respectively. $\mathcal{H}_{ij}$ can be rewritten as

$$\begin{aligned}
\mathcal{H}_{12} &= J\mathbf{S}_1 \cdot \mathbf{S}_2 + \Delta\mathcal{J} S_{1,z} \cdot S_{2,z} + \mathbf{d}_{12} \cdot (\mathbf{S}_1 \times \mathbf{S}_2) - \mathcal{J}_3\big(S_{1,x}S_{2,y} + S_{1,y}S_{2,x}\big), \\
\mathcal{H}_{13} &= J\mathbf{S}_1 \cdot \mathbf{S}_3 + \Delta\mathcal{J} S_{1,y} \cdot S_{3,y} + \mathbf{d}_{13} \cdot (\mathbf{S}_1 \times \mathbf{S}_3) - \mathcal{J}_3\big(S_{1,x}S_{3,z} + S_{1,z}S_{3,x}\big), \\
\mathcal{H}_{14} &= J\mathbf{S}_1 \cdot \mathbf{S}_4 + \Delta\mathcal{J} S_{1,x} \cdot S_{4,x} + \mathbf{d}_{14} \cdot (\mathbf{S}_1 \times \mathbf{S}_4) + \mathcal{J}_3\big(S_{1,y}S_{4,z} + S_{1,z}S_{4,y}\big), \\
\mathcal{H}_{23} &= J\mathbf{S}_2 \cdot \mathbf{S}_3 + \Delta\mathcal{J} S_{2,x} \cdot S_{3,x} + \mathbf{d}_{23} \cdot (\mathbf{S}_2 \times \mathbf{S}_3) - \mathcal{J}_3\big(S_{2,y}S_{3,z} + S_{2,z}S_{3,y}\big), \\
\mathcal{H}_{24} &= J\mathbf{S}_2 \cdot \mathbf{S}_4 + \Delta\mathcal{J} S_{2,y} \cdot S_{4,y} + \mathbf{d}_{24} \cdot (\mathbf{S}_2 \times \mathbf{S}_4) + \mathcal{J}_3\big(S_{2,x}S_{4,z} + S_{2,z}S_{4,x}\big), \\
\mathcal{H}_{34} &= J\mathbf{S}_3 \cdot \mathbf{S}_4 + \Delta\mathcal{J} S_{3,z} \cdot S_{4,z} + \mathbf{d}_{34} \cdot (\mathbf{S}_3 \times \mathbf{S}_4) + \mathcal{J}_3\big(S_{3,x}S_{4,y} + S_{3,y}S_{4,x}\big),
\end{aligned} \tag{10}$$

where $J = \mathcal{J}_1$, $\Delta\mathcal{J} = \mathcal{J}_2 - \mathcal{J}_1$, $\mathbf{d}_{ij} = \sqrt{2}\mathcal{J}_4\hat{\mathbf{d}}_{ij}$. The DM vector directions $\hat{\mathbf{d}}_{ij}$ between sites *i* and *j* (see the inset of Fig. 1c) are

$$\begin{aligned}
&\hat{\mathbf{d}}_{12} = (1,1,0)/\sqrt{2}\,, \hat{\mathbf{d}}_{13} = (-1,0,-1)/\sqrt{2}\,, \hat{\mathbf{d}}_{14} = (0,-1,1)/\sqrt{2}, \\
&\hat{\mathbf{d}}_{23} = (0,1,1)/\sqrt{2}\,, \hat{\mathbf{d}}_{24} = (1,0,-1)/\sqrt{2}\,, \hat{\mathbf{d}}_{34} = (-1,1,0)/\sqrt{2}\,.
\end{aligned} \tag{11}$$

Fitting the inelastic neutron scattering data with six parameters $\mathcal{J}_1$, $\mathcal{J}_2$, $\mathcal{J}_3$, $\mathcal{J}_4$, $g_\parallel$ and $g_\perp$ (see Supplementary Note 4), we obtained optimized values of $\mathcal{J}_1 = 0.5872(4)$ meV, $\mathcal{J}_2/\mathcal{J}_1 = 0.987(2)$, $\mathcal{J}_3/\mathcal{J}_1 = -0.013(1)$, $\sqrt{2}\mathcal{J}_4/\mathcal{J}_1 \equiv d_{ij}/J = 0.2713(9)$, $g_\parallel = 2.707(3)$, and $g_\perp = 2.365(3)$, showing that $\mathcal{J}_2 \approx \mathcal{J}_1$ ($\Delta\mathcal{J} \approx 0$) and $\mathcal{J}_3 \approx 0$. Therefore, one can notice that the generalized Hamiltonian is reduced to an effective exchange Hamiltonian with a DM interaction as

$$\mathcal{H}_{\mathrm{eff}} = J\sum_{i<j}\mathbf{S}_i \cdot \mathbf{S}_j + \sum_{i<j}\mathbf{d}_{ij} \cdot \big(\mathbf{S}_i \times \mathbf{S}_j\big) - \mu_B\mathbf{H} \cdot \sum_i \mathbf{g}_i \cdot \mathbf{S}_i\,. \tag{12}$$

## Supplementary Note 2. *g*-factors: Electron paramagnetic resonance

In order to determine the $g$-factors, we performed high-frequency electron paramagnetic resonance (EPR) measurements at $\nu$ = 104 GHz using the transmission spectrometer developed at the National High Magnetic Field Laboratory with a sweepable 15-T superconducting magnet. Supplementary Fig. 4 shows the EPR spectrum of the $Ba_3Yb_2Zn_5O_{11}$ powder sample measured at $T$ = 295 K. The spectrum is composed of two peaks, which is well simulated with the two effective $g$-values, $g_{\parallel} = 2.54$ and $g_{\perp} = 2.13$ and the peak-to-peak linewidths, $\Delta H_{pp}$ = 197.9(6) mT and 434.3(7) mT (see the solid red line). The observed Lorentzian line-shape means that the EPR signal is exchange-narrowed due to fast electronic fluctuations of $Yb^{3+}$ ions through an antiferromagnetic exchange interaction. We note that the experimentally determined $g$-values are not significantly different from those evaluated by the crystal field model calculation $g_{\parallel} = 2.87$ and $g_{\perp} = 2.27$ (see Supplementary Note 4).

## Supplementary Note 3. Magnetization

The effective Hamiltonian $\mathcal{H}_{\mathrm{eff}}$ consisting of the Heisenberg interaction, the DM interaction and the anisotropic Zeeman term (see Supplementary Note 1) enables us to explain three characteristic features of the field dependent magnetization *M*(*H*) such as step-like jumps at $H_{C1}$ = 3.5 T and $H_{C2}$ = 8.8 T, non-zero slope below $H_{C1}$, and hysteresis near $H_{C1}$. The DM interaction [5] and the anisotropic Zeeman term, which are non-commutative with the Heisenberg interaction term and then reconstruct eigenstates of the conventional Heisenberg Hamiltonian, not only affects level crossing critical fields ($H_{C1}$ and $H_{C2}$) but also drives a paramagnetic response to applied magnetic field below $H_{C1}$. Supplementary Fig. 5 shows three simulated *M*(*H*)'s from $\mathcal{H}_{\mathrm{eff}}$ under an adiabatic process, which are compared to the measured ones with the lowest rate (7.5 mT·min$^{-1}$) in magnetic field-up and –down sweeps. No DM interaction (*d*/*J* = 0) with an isotropic *g*-factor (*g* = 2.569), no DM with anisotropic g-factors ($g_{\parallel} = 3.0$ and $g_{\perp} = 2.4$), and the DM with anisotropic *g*-factors (*d*/*J* = 0.27, $g_{\parallel} = 3.0$ and $g_{\perp} = 2.4$) are represented by a black dashed, a cyan dashed and a green solid line, respectively. Among three models, the last one (the green line) gives the best simulation. It is noted that the effect of DM on the *M*(*H*) in $0 < H < H_{C1}$ is twice bigger than that of the anisotropic Zeeman term as shown in the inset of Supplementary Fig. 5, which indicates that the DM interaction admixes the $S_{\mathrm{eff}}$ = 0 single states and $S_{\mathrm{eff}}$ = 1 triplet states more significantly.

The simulated *M*(*H*) well reproduces overall features except the hysteretic behavior related to a finite field sweep rate (non-adiabatic process). This hysteretic behavior results from the non-equilibrium Landau-Zener transition, which involves a two-level system with avoided level crossing [6, 7]. As the level energies vary with the magnetic field as shown in the inset of Fig. 1d, the transition probability between the two levels can be described by $P = 1 - \exp(-\pi\Delta_0^2/4\hbar\mu_{\mathrm{B}}r)$ with the avoided level crossing energy gap $\Delta_0$ and the field sweep rate *r*. As the sweep rate *r* goes to zero (adiabatic process), the transition probability becomes 1 and the hysteretic behavior disappears since the level occupations are simply determined by the Boltzmann factor. Meanwhile, as *r* increases, the probability decreases and the hysteretic behavior becomes noticeable. Indeed, one can recognize that the hysteretic behavior becomes intensified in *M*(*H*) with a faster sweep rate (30 mT·min$^{-1}$) as shown in Supplementary Fig. 6.

## Supplementary Note 4. $YbO_6$ crystal field analyses and magnetic exchange coupling constants

The crystal field (CF) splittings of Yb 4*f* in $Ba_3Yb_2Zn_5O_{11}$ can be estimated by using a simple point charge model under the $C_{3\mathrm{v}}$ local symmetry of a distorted $YbO_6$ octahedron. The large spin orbit coupling energy $\zeta$ splits the atomic 4*f* level into the total angular momentum $J_{7/2}$ and $J_{5/2}$ states. In $Yb^{3+}$ ($4f^{13}$), the low lying $J_{5/2}$ state is fully occupied and one hole is left in the high lying $J_{7/2}$ state. The splitting energy $\eta = 7\zeta/2$ is sufficiently large in comparison with the 4*f* crystal field splitting energy so that we neglect the $J_{5/2}$ states. The 8-fold $J_{7/2}$ state is split into two doublets and a quartet by the $O_h$ CF, and finally into four doublets (Kramers doublets) under the $C_{3\mathrm{v}}$ local symmetry as schematically depicted in Supplementary Fig. 2.

For estimation of the level splittings, the ionic positions are referred to the neutron powder diffraction results with Rietveld refinements. The CF Hamiltonian under the $C_{3\mathrm{v}}$ symmetry can be described by six CF terms,

$$\mathcal{H}_{\mathrm{CEF}} = B_2^0 O_2^0 + B_4^0 O_4^0 + B_4^3 O_4^3 + B_6^0 O_6^0 + B_6^3 O_6^3 + B_6^6 O_6^6, \tag{13}$$

where $B_m^n$ are effective CF parameters, and $O_m^n$ are the Steven's operators. $B_m^n$ can be decomposed as $B_m^n = \langle r^m \rangle \sum_i \frac{1}{d_i^{m+1}} A_m^n(\theta_i, \varphi_i)$, where $\langle r^m \rangle$ are expectation values of 4*f* electrons and $(d_i, \theta_i, \varphi_i)$ denotes the position of *i*-th oxygen in the $YbO_6$ octahedron.

In the ground state, one 4f hole of $Yb^{3+}$ ion is introduced to the highest Kramers doublet states,

$$|\pm\rangle_{z_i} = \mp C_1 \left|\tfrac{7}{2}, \mp\tfrac{7}{2}\right\rangle_{z_i} - C_2 \left|\tfrac{7}{2}, \mp\tfrac{1}{2}\right\rangle_{z_i} \pm C_3 \left|\tfrac{7}{2}, \pm\tfrac{5}{2}\right\rangle_{z_i}, \tag{14}$$

as described Supplementary Note 1. By using CF parameter values of $B_2^0 = 0.577, B_4^0 = 1.643 \times 10^2, B_4^3 = -3.902 \times 10^3, B_6^0 = 3.826 \times 10^4, B_6^3 = 3.596 \times 10^6, B_6^6 = 9.175 \times 10^5$, we estimated the CF splitting energies $E_1$ = 35.9 meV, $E_2$ = 54.5 meV, and $E_3$ = 68.3 meV, which are respectively the energy separations of the first, second, and third excited doublets from the ground doublet as denoted in Supplementary Fig. 2. These splitting energies well agree with the experimental values within error bars, which are obtained from high energy neutron excitation spectra [2]. Based on the CF model calculation results, one can determine the ground state Kramer doublet states with $C_1$ = 0.0983, $C_2$ = 0.6287, and $C_3$ = 0.7714, which yield the *g*-factors values of $g_{\parallel} = 2.87$ and $g_{\perp} = 2.27$.

Using the Kramers doublet states, we now examine the magnetic exchange coupling constants $\mathcal{J}_i$ (*i*= 1, 2, 3, and 4) in the tensor $\mathbf{J}$ as presented in Supplementary Note 1. Considering two edge-shared Yb ions at sites 3 and 4 (see Supplementary Fig. 3), the hopping Hamiltonian, which is spin-independent, is expressed as

$$\mathcal{H}_{\mathrm{t}} = \sum_{\alpha\beta\sigma} t_{\alpha\beta} \left( c_{3\alpha\sigma}^{\dagger} c_{4\beta\sigma} + c_{4\beta\sigma}^{\dagger} c_{3\alpha\sigma} \right), \tag{15}$$

where $c_{i\alpha\sigma}$ is an annihilation operator of 4*f* $\alpha$-orbital with a $\sigma$ spin at the *i*-th site. The *f*-*f* intersite hopping is completely dominated by the superexchange hopping through ligand *p* orbitals. According to the Slater-Koster theory [8], the edge-shared geometry yields non-zero values in the hopping matrix $t_{\alpha\beta}$ only for

$t_{z^3,z^3} = -\frac{3}{4}t$ and $t_{z(x^2-y^2),z(x^2-y^2)} = \frac{5}{4}t$ with $t = V_{pf\pi}^2/\Delta_{\mathrm{CT}}$ . $V_{pf\pi}$ denotes the $2p$-$4f$ $\pi$-bonding hybridization strength and $\Delta_{\mathrm{CT}}$ is the charge transfer energy.

Confining the Kramers doublet for the Yb site, we can only consider four states of the two Yb site cluster for the hopping. Those states are $|\Psi_1\rangle = |D_+\rangle_{z_3} \otimes |D_+\rangle_{z_4}$, $|\Psi_2\rangle = |D_+\rangle_{z_3} \otimes |D_-\rangle_{z_4}$, $|\Psi_3\rangle = |D_-\rangle_{z_3} \otimes |D_+\rangle_{z_4}$ , and $|\Psi_4\rangle = |D_-\rangle_{z_3} \otimes |D_-\rangle_{z_4}$ . In the limit of the second-order perturbation, its effective Hamiltonian is given by

$$\mathcal{H}_{\mathrm{t,eff}}^{(34)} = \sum_{1\le n,n'\le 4} \sum_m \frac{\langle \Psi_n | H_t | \Psi_m^{eh} \rangle \langle \Psi_m^{eh} | H_t | \Psi_{n'} \rangle}{E_0 - E_m} |\Psi_n\rangle\langle\Psi_{n'}| = \sum_{1\le n,n'\le 4} \mathcal{H}_{nn'}^{(34)} |\Psi_n\rangle\langle\Psi_{n'}| , \quad (16)$$

where $|\Psi_m^{eh}\rangle$ refer to unperturbed excited states with energy $E_m$ , which are overlapped with $|\Psi_n\rangle$ through the superexchange hopping. For simplicity, we adopt a fixed $U = E_m - E_0$ for all $|\Psi_m^{eh}\rangle$. Then the $\mathcal{H}^{(34)}$ matrix can be expressed in terms of the exchange coupling matrix elements $\mathcal{J}'_{\mu\nu}$ ($\mu, \nu = x, y, z$) in the local coordinates as defined in Supplementary Note 1:

$$\mathcal{H}^{(34)} = CI + \frac{1}{4}\begin{pmatrix} \mathcal{J}'_{zz} & \mathcal{J}'_{zx} - i\mathcal{J}'_{zy} & \mathcal{J}'_{xz} - i\mathcal{J}'_{yz} & \mathcal{J}'_{xx} - \mathcal{J}'_{yy} - i(\mathcal{J}'_{xy} + \mathcal{J}'_{yx}) \\ & -\mathcal{J}'_{zz} & \mathcal{J}'_{xx} + \mathcal{J}'_{yy} + i(\mathcal{J}'_{xy} - \mathcal{J}'_{yx}) & -\mathcal{J}'_{xz} + i\mathcal{J}'_{yz} \\ & & -\mathcal{J}'_{zz} & -\mathcal{J}'_{zz} + i\mathcal{J}'_{zy} \\ & & & \mathcal{J}'_{zz} \end{pmatrix}, \quad (17)$$

where the lower off-diagonal matrix elements correspond to the Hermitian conjugates of the upper off-diagonal ones. By making the unitary transformation to the global coordinates as described in Supplementary Note 1, the exchange coupling tensor is reduced to $\mathbf{J}_{34}$,

$$\mathbf{J}_{34} = \begin{pmatrix} \mathcal{J}_1 & \mathcal{J}_3 & -\mathcal{J}_4 \\ \mathcal{J}_3 & \mathcal{J}_1 & -\mathcal{J}_4 \\ \mathcal{J}_4 & \mathcal{J}_4 & \mathcal{J}_2 \end{pmatrix}. \quad (18)$$

Applying the CF model calculation results, $C_1$ = 0.0983, $C_2$ = 0.6287, and $C_3$ = 0.7714, for the Kramers doublet states, we obtained the exchange coupling matrix,

$$\mathbf{J}_{34} \approx \frac{t^2}{U}\begin{pmatrix} 0.2123 & -0.0042 & -0.0422 \\ -0.0042 & 0.2123 & -0.0422 \\ 0.0422 & 0.0422 & 0.2081 \end{pmatrix}, \quad (19)$$

resulting in $d/J$ = 0.281, which agrees well with the ratio $d/J$ = 0.27 determined from the inelastic neutron scattering data.

## Supplementary References


[1] Onoda, S. Effective quantum pseudospin-1/2 model for Yb pyrochlore oxides, *J. Phys.; Conf. Ser.* **320**, 012065 (2011).

[2] Haku, T. *et al.* Crystal Field Excitations in the Breathing Pyrochlore Antiferromagnet $Ba_3Yb_2Zn_5O_{11}$. *J. Phys. Soc. Jpn*. 85, 034721 (2016).

[3] McClarty, P. A., Curnoe, S. H. & Gingras, M. J. P. Energetic selection of ordered states in a model of the $Er_2Ti_2O_7$ frustrated pyrochlore XY antiferromagnet. *J. Phys.: Conference Series* **145**, 012032 (2009).

[4] Ross, K. A., Savary, L., Gaulin, B. D. & Balents, L. Quantum Excitations in Quantum Spin Ice, *Phys. Rev. X* **1**, 021002 (2011).

[5] Kotov., V. N., Zhitomirsky, M. E., Elhajal, M. & Mila, F. Weak antiferromagnetism and dimer order in quantum systems of coupled tetrahedra. *Phys. Rev. B* **70**, 214401 (2004).

[6] Chiorescu., I, Wernsdorfer, W., Müller, A., Bögge, H. & Barbara, B. Butterfly Hysteresis Loop and Dissipative Spin Reversal in the $S$ = 1/2, $V_{15}$ Molecular Complex. *Phys. Rev. Lett.* **84**, 3454-3457 (2000).

[7] Waldmann, O., Koch, R., Schromm, S., Müller, P., Bernt, I. & Saalfrank, R. W. Butterfly Hysteresis Loop at Nonzero Bias Field in Antiferromagnetic Molecular Rings: Cooling by Adiabatic Magnetization. *Phys. Rev. Lett.* **89**, 246401 (2002).

[9] Slater, J. C., Koster, G. F. Simplified LCAO Method for the Periodic Potential Problem, *Phys. Rev.* **94**, 1498-1524 (1954).
